# Exploring functional roles of TRPV1 intracellular domains with unstructured peptide-insertion screening

**DOI:** 10.1038/srep33827

**Published:** 2016-09-26

**Authors:** Linlin Ma, Fan Yang, Simon Vu, Jie Zheng

**Affiliations:** 1 Department of Physiology and Membrane Biology, University of California School of Medicine, Davis CA 95616, USA; 2 Institute for Molecular Bioscience, University of Queensland, St Lucia, QLD 4072, Australia

## Abstract

TRPV1 is a polymodal nociceptor for diverse physical and chemical stimuli that interact with different parts of the channel protein. Recent cryo-EM studies revealed detailed channel structures, opening the door for mapping structural elements mediating activation by each stimulus. Towards this goal, here we have combined unstructured peptide-insertion screening (UPS) with electrophysiological and fluorescence recordings to explore structural and functional roles of the intracellular regions of TRPV1 in mediating various activation stimuli. We found that most of the tightly packed protein regions did not tolerate structural perturbation by UPS when tested, indicating that structural integrity of the intracellular region is critical. In agreement with previous reports, Ca^2+^-dependent desensitization is strongly dependent on both intracellular N- and C-terminal domains; insertions of an unstructured peptide between these domains and the transmembrane core domain nearly eliminated Ca^2+^-dependent desensitization. In contrast, channel activations by capsaicin, low pH, divalent cations, and even heat are mostly intact in mutant channels containing the same insertions. These observations suggest that the transmembrane core domain of TRPV1, but not the intracellular domains, is responsible for sensing these stimuli.

TRPV1 ion channel can be directly activated or modulated by capsaicin^1^, heat^1^, extracellular proton^2^, divalent cations^3^, Na^+ ^4, peptide toxins from spider^5^, centipede^6^, and scorpion^7^, membrane depolarization^8^, intracellular Ca^2+^ 9, and many other factors^10^. A noticeable feature of TRPV1 polymodal activation emerging from biophysical investigations is the existence of non-overlapping activation pathways^11^,^12^,^13^. However, except for capsaicin^14^,^15^,^16^,^17^,^18^,^19^, proton^11^,^20^, and animal toxins^6^,^21^, the channel structures that sense activation stimuli are poorly defined. Recent cryo-EM studies revealed that the transmembrane core domain of TRPV1 resembles that of tetrameric cation channels, with six transmembrane helical segments surrounding a centrally located ion permeation pore^22^,^23^. The partially resolved intracellular N- and C-terminal domains contain special structures such as the ankyrin-like repeat domains and the TRP domain that likely play crucial structural and/or functional roles^10^. Arrangement of intracellular domains has been investigated using fluorescence resonance energy transfer^24^. A major task for TRPV1 study in the post-structure era is to assign functional roles to individual structure domains.

The unstructured peptide-insertion screening (UPS) strategy had been used to rapidly and reliably obtain information on structure-function relationships in an ion channel even before detailed protein structures were available. In one effective application, isolating the pore domain of yeast TRPY1 channel from the intracellular Ca^2+^-sensing domain with unstructured peptides demonstrated that the pore domain was mechanosensitive^25^. Similarly, unstructured peptide insertion was used to perturb functional coupling between channel pore and the intracellular Ca^2+^-binding domains of the BK channel^26^. More recently, this strategy was applied to the study of TREK-1 potassium channel, which is highly temperature-sensitive like TRPV1^27^. Introducing an unstructured triple-glycine peptide between the M4 segment and the C terminus was found to eliminate high temperature-sensitivity, supporting the idea that the C-terminus of TREK-1 is responsible for sensing temperature change^28^. In these studies, insertion of a short, unstructured peptide was used to perturb coupling between two rigid channel domains. With the guidance of detailed structural information, unstructured peptides can also be introduced into a rigid domain to disrupt its structural integrity and test its functional contribution. In the present study, we applied the UPS approach to TRPV1 intracellular domains to explore their structural role and functional contribution to polymodal activation.

## Materials and Methods

### Molecular biology and transfection

The mouse TRPV1 was used in this study; its amino acid numbers differ from the rat TRPV1, another commonly used version, mostly by one—for example, Y511 in mTRPV1 corresponds to Y512 in rTRPV1. Channel cDNA was constructed into mammalian expression vector pEYFP-N3 as described previously^29^, which led to an eYFP tag fused in frame to the C-terminal end of the mTRPV1 cDNA. Site-directed insertion mutagenesis was performed using either QuikChange II mutagenesis kit (Stratagene) according to the manufacturer’s instruction or the standard overlap extension PCR method. All mutations were verified by sequencing. HEK293 cells were cultured in a DMEM medium supplemented with 10% FBS, 2 mM glutamine, 1% (v/v) non-essential amino acids, at 37 °C with 5% CO_2_. Cells were plated on 0.1 mg/ml poly-D-lysine coated glass coverslips 24 h before transfection to improve cell adhesion during subsequent imaging and patch recordings. HEK293 cells were transfected with Lipofectamine 2000 (Invitrogen) following standard protocols. Imaging studies and patch-clamp recordings were performed 24-to-48 h after transfection.

### Confocal imaging

Cellular distribution of expressed TRPV1 wild-type and mutants were examined using confocal microscopy. The Airyscan mode of Zeiss LSM880 controlled by the ZEN software (version 2.1 black) was used to achieve super-resolutions up to 140 nm. Laser irradiation at 458 nm and 514 nm was used to excite eYFP linked to the channel and the plasma membrane marker di-8-ANEPPS, respectively. A 40X oil immersion lens with a 1.30 numerical aperture was used. eYFP was imaged with a main beam splitter at 458/514 nm and a 495–550 nm band pass emission filter. Di-8-ANEPPS was imaged with a main beam splitter at 458 nm and a 570 nm long pass emission filter.

### Calcium imaging

Transiently transfected HEK293 cells seeded on 25 mm coverslips were washed twice with an extracellular solution (ECS) containing 140 mM NaCl, 5 mM KCl, 1 mM MgCl_2_, 1.8 mM CaCl_2_, 10 mM glucose, and 15 mM HEPES (pH 7.4), followed by incubation in 2 ml of ECS supplemented with 2 μM Fluo-4/AM (Kd for Ca^2+^ at 345 nM) and 0.05% Pluronic F-127 (both from Molecular Probes) at room temperature for 60 min. Probenecid (2 mM) was included in all solutions to prevent Fluo-4 leakage from cells. At the end of incubation, cells were washed three times with ECS and incubated in the same solution for another 20 min at room temperature to complete the intracellular hydrolysis process of the AM ester, which converts the non-fluorescent Fluo-4/AM into the fluorescent version Fluo-4.

Coverslip with dye-loaded cells was placed in the quick-release magnetic chamber (Warner) and mounted on the stage of a Nikon Eclipse TE2000-U microscope system equipped with a Roper Cascade 128B CCD camera. Fluo-4 was excited by an Argon laser with a filter set of z488/10 (excitation), z488rdc (dichroic) and recorded through an emission filter HQ500lp (all from Chroma). The duration of light exposure was controlled by a computer-driven mechanical shutter (Uniblitz). Cell images were acquired sequentially with an exposure period of 200 ms at an interval of 1 s. The shutter and the camera were controlled and synchronized by MetaMorph software (Universal Imaging). Cells pretreated with 1 μM thapsigargin during the dye-loading step (aiming to deplete ER Ca^2+^ store) did not exhibit noticeable difference in fluorescence intensity or kinetics compared to untreated cells. Change in fluorescence intensity, ΔF, was calculated as the difference between the equilibrium level before and after stimulation.

### Electrophysiology

Patch-clamp recordings were performed with an EPC10 amplifier (HEKA) driven by PatchMaster software (HEKA). Patch pipettes were pulled from borosilicate glass and fire-polished to a resistance of 2–5 МΩ. The membrane potential was held at 0 mV, and currents were elicited by a protocol consisting of a 300-ms step to +80 mV followed by a 200-ms step to −80 mV at 1-s intervals. The steady-state current amplitude was normally measured at +80 mV. For inside-out or outside-out patch recordings, both pipette solution and bath solution contained 130 mM NaCl, 0.2 mM EDTA, and 3 mM Hepes (pH 7.2). In experiments recording Ca^2+^-dependent desensitization of TPRV1 using whole-cell patch configuration, 2 mM CaCl_2_ was added to both solutions and EGTA was omitted. Current signals were filtered at 2.9 kHz and sampled at 10 kHz. Solution switching was achieved with a rapid solution changer RSC-200 (Biological Science Instruments).

### Temperature control

Temperature control for both calcium imaging and patch clamping was achieved by perfusion of preheated solutions as described previously^13^. Briefly, solutions were heated with an SHM-828 eight-line heater controlled by a CL-100 temperature controller (Harvard Apparatus). The patch pipette was placed about 1 mm from the solution output ports. Local temperature was accurately monitored by a BAT-12 thermometer with ultrafine thermocouple probe (Physitemp) placed right next to the pipette. The thermometer’s temperature readout was fed into an analog input of the patch amplifier and recorded simultaneously with current in patch clamp recordings. For calcium imaging, MetaMorph software was synchronized with PatchMaster software to record temperature simultaneously with imaging. When the experimental temperature was not controlled, recordings were conducted at room temperature at ~24 °C.

### Data analysis

Analysis of electrophysiological data was done with PatchMaster and Igor. When necessary, a digital filter was applied to the saved current traces. The capsaicin concentration-dependent activation curves were fit to a Hill equation to obtain the EC50 value and the slope factor. Imaging analysis was done with MetaMorph.

Heat sensitivity was evaluated with two approaches. In the first approach, the slope of heat-dependent current activation was quantified by an R value that was calculated as R = (I_2_/I_1_)^(10/(T_2_−T_1_))^, in which T_1_ is the threshold temperature, T_2_ = T_1_ + 5 °C, and I_1_ and I_2_ are current amplitude at T_1_ and T_2_, respectively^30^. In the second approach, current activated by heating to 43 °C was normalized to the peak current activated by 10 μM capsaicin at RT and designated as I_heat_/I_Cap_. When the current started to inactivate before reaching 43 °C, I_heat_ was measured at the peak current, resulting in an underestimate of the relative amplitude of the heat-induced activation.

To quantify the temperature threshold for heat activation, the current raising phase was plotted as a function of temperature. The current-temperature relationship of TRPV1 usually exhibited two major phases. The first slow phase represented mostly temperature-dependent increase in the leak current (R value of ~1.7), whereas the second rapid takeoff phase represented heat-induced TRPV1 channel activation. The activation threshold temperature was defined as the intersect point of liner fits to these two phases^30^.

### Rosetta structural modeling

Homology-membrane-symmetry-loop modeling of the pore-forming domain of TRPV1 channel was performed using the Rosetta method^31^. Coordinates of S1-S5, Pore helix, and S6, were taken from TRPV1 channel structures (PDB ID: 3J5P and 3J5R) and kept rigid during modeling. The loop regions between S2 and S3 in each subunit of the tetramer were modeled *de novo*. During the first round of modeling, Rosetta’s cyclic coordinate descent (CCD) loop relax protocol^32^ was used and the top 20 cluster center models were passed to the second round. During the second to third rounds of modeling, kinematic loop relax protocol (KIC)^33^ was used and the top 20 cluster center models were passed to the next round. During the rest rounds of modeling, kinematic loop relax protocol was used and the top 20 models by score were passed to the next round. From 10,000 to 20,000 models were generated in each round. The models shown here represent the 20 lowest energy models from the last round of iterative loop relax.

### Statistics

All statistical data are given as mean ± SEM. Student’s *t*-test was applied to examine the statistical significance. *, **, ***, and n.s. denote P < 0.05, P < 0.01, and P < 0.001, and not significant, respectively.

## Results

### Screening TRPV1 intracellular domains with unstructured peptide insertion

**Figure 1:**
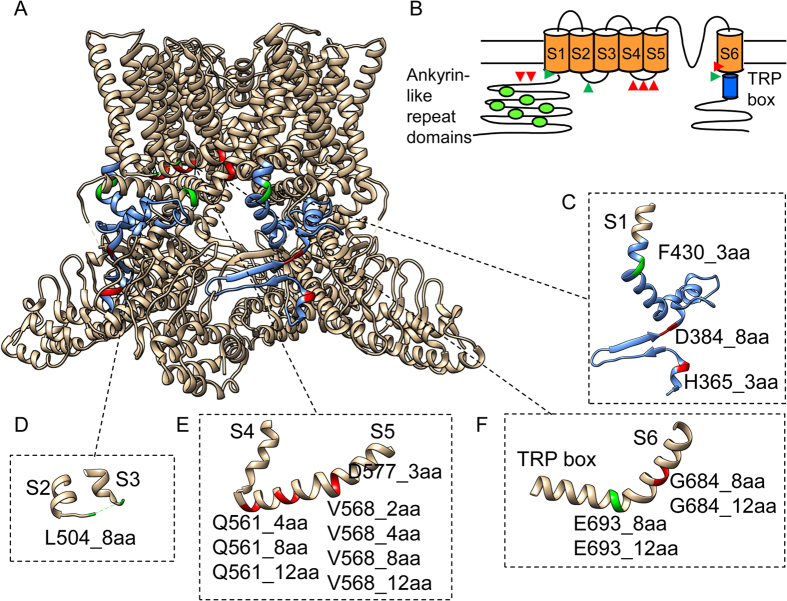
Unstructured peptide-insertion screening. (**A**) TRPV1 residues bracketing the insertion sites are highlighted in green (functional) and red (non-functional). The linker between the ankyrin-like repeat domain and S1 is shown in blue. (**B**) A schematic diagram showing the membrane topology of a TRPV1 subunit. Insertion sites that yield functional and non-functional mutants are marked by green and red triangles, respectively. (**C***–***F**) Zoom-in view of the channel structures containing peptide insertions. Insertions are named in the format of Xnnn_maa, in which X represents the single-letter amino acid preceding the insertion, nnn represents the residue number, m represents the number of amino acids in the inserted peptide.

**Figure 2:**
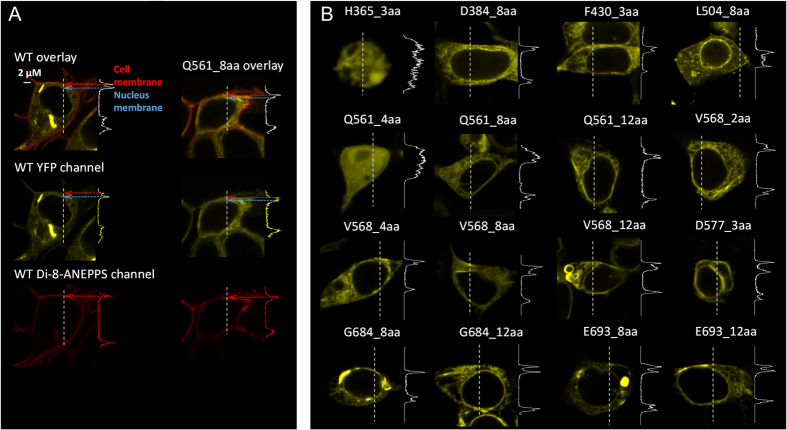
Confocal imaging of cellular distribution of expressed channel proteins. (**A**) Images of a HEK293 cell expressing the wild-type TRPV1_eYFP (left three panels) reveal substantial overlap between eYFP (yellow) and the plasma membrane dye di-8-ANEPPS (red). Images of cells expressing a mutant channel (Q561_8aa) are shown in the right panel. The fluorescence intensity profile from a line scan (indicated by dash line) is shown on the right side. (**B**) Representative images of mutant-expressing cells and the fluorescence intensity profile.

Guided by the TRPV1 cryo-EM structures^22^,^23^, we have identified a series of intracellular sites for UPS tests (Fig. 1A,B). Our strategy was to focus on key structural domains as well as junctions between domains. After expressing the sixteen insertion mutants in HEK293 cells, we first conducted confocal imaging to examine the distribution of eYFP-tagged channel proteins. While it is well-known that transient over-expression often leads to a large amount of protein trapped inside the cell, we observed in most cases clear plasma membrane distribution of fluorescence (Fig. 2). To confirm the location of fluorescence signal, a plasma membrane marker, di-8-ANEPPS (a gift from the Santana lab), was used to label the same cells (Fig. 2A). Overlap of channel-eYFP and di-8-ANEPPS signals was seen only at the plasma membrane, suggesting proper trafficking of the mutant channels. Two exceptions, H365_3aa and Q561_4aa, were identified, for which the fluorescence signal was evenly distributed across the cell (Fig. 2B).

### Functional tests of insertion mutants

**Figure 3:**
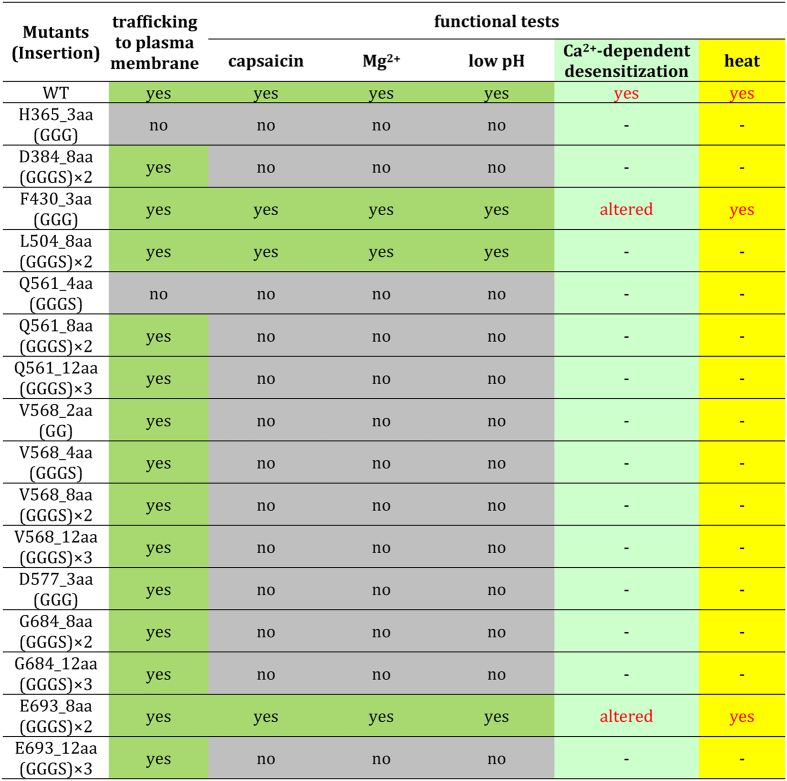
Properties of UPS mutants.

We conducted both Ca^2+^-imaging and patch-clamp recording from each of the insertion mutants. A summary of these characterizations is provided below and in Fig. 3, followed by detailed discussions of key insertions.

#### The N-terminal region

TRPV1 N-terminus contains an ankyrin-like repeat domain whose crystal structure and functional role have been subjected to intensive investigations^34^,^35^,^36^. The ankyrin-like repeat domain is linked to the first transmembrane segment (S1) by a 77-amino-acid segment thought to be the heat sensor^37^ (colored blue in Fig. 1A,C). A noticeable structural element within this segment is the anti-parallel β-hairpin pointing to the ankyrin-like repeat domain of a neighboring subunit (Fig. 1C). It is anticipated that this unique structure may provide crucial coupling between adjacent subunits^22^. To test this attractive idea, we inserted a short unstructured peptide either before or after the β-hairpin. Functional tests revealed that channel-expressing cells did not respond to 10 μM capsaicin, pH 4.6, or 130 mM Mg^2+^, indicating that the mutant channels were non-functional. Our observations are supportive of a crucial structural/functional role of the β-hairpin structure.

Another insertion mutation was made at the junction between the N-terminus and S1 (Fig. 1A,C, green) that, together with the TRP domain (discussed below), couples intracellular domains to the transmembrane core domain. To perturb this coupling, a triple-glycine peptide was introduced at the turn between S1 and its preceding helical segment. We found that this mutant channel, named F430_3aa, was functional.

#### S2-S3 linker

The structure of S2-S3 linker was not modeled in the previous cryo-EM study, though the corresponding electron density of this linker was clearly observed^22^. When an eight-amino-acid peptide was inserted here (Fig. 1D), the channel, named L504_8aa, was functional.

#### S4-S5 linker

The S4-S5 linker forms a horizontal helix connecting the peripheral S1-S4 domain to the central pore domain^22^. Recent studies confirmed that it serves a critical role in coupling capsaicin binding to channel activation^16^,^17^,^18^,^19^. We made a series of insertions of 2-to-12 amino acids in length into this key structure, at three different positions (Fig. 1E). Functional tests however showed that none of these channels could respond to capsaicin, low pH, or Mg^2+^, though the proteins appeared to be expressed in plasma membrane (Fig. 2B). Therefore, it is clear that a rigid structure of S4-S5 linker is required for channel function.

#### The C-terminal region

Except for two isolated segments, the structure for most of the C-terminus is unavailable^22^. One of the resolved structures is a short β-strand that runs parallel to the β-hairpin mentioned above to form a β-sheet between adjacent subunits. Our insertion experiments on the N-terminal side (Fig. 1C) already indicated that this unique structure plays an important role in channel structure/function. The other resolved structure is the anterior C-terminal segment right after S6. Here the TRP domain is conserved among many TRP channels and key to channel function^38^,^39^. This segment forms a continuous helix that is sandwiched between the S4-S5 linker and pre-S1 segment^22^. We made four insertions at two separate positions (Fig. 1F), out of which only one, named E693_8aa, was functional. The insertion was made at the “turn” between S6 and the TRP domain, where the structure exhibits the relaxed π-helix character. Interestingly, insertion of a longer (12 amino acids) peptide at this same position resulted in non-functional channel. Likewise, the two insertions within the helical structure of S6 resulted in non-functional channels.

### No noticeable effect of functional insertions on channel activation by capsaicin, low pH, or Mg^2+^

**Figure 4:**
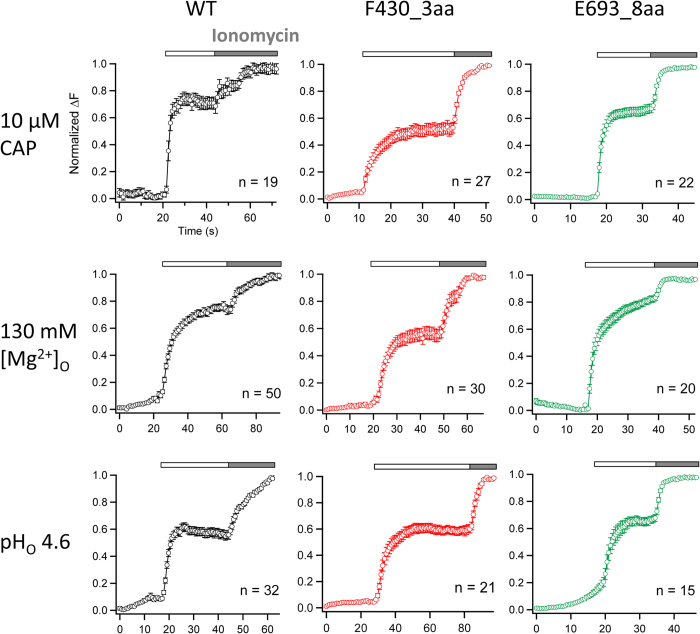
Inserting an unstructured peptide between the transmembrane core domain and the intracellular domains does not affect general channel function. Averaged calcium imaging recordings of wild-type (WT) TRPV1 and two insertion mutants of interest, F430_3aa and E693_8aa, activated by extracellularly applied (as indicated by white bars) 10 μM capsaicin, 130 mM Mg^2+^ and low pH (pH 4.6) respectively. 3 mM ionomycin (as indicated by gray bars) was applied at the end of each imaging experiment to get the maximum fluorescence change. The total number of cells tested is given in each panel. The error bar represents standard error of means. Change in fluorescence intensity, *ΔF,* was calculated as the difference between the equilibrium level before stimulus application and after application of ionomycin.

To assess the impact of unstructured peptide insertion at intracellular sites to channel function, we used live-cell fluorescence imaging to record intracellular Ca^2+^ increases upon activation of TRPV1, a non-selective cation channel with high Ca^2+^-permeability^1^. Control experiment with cells expressing the wild-type channels showed that, upon adding capsaicin, there was a rapid increase of the intracellular fluorescence intensity that reached peak level in a couple of seconds (Fig. 4). No increase in fluorescence intensity could be observed from untransfected cells, or cells over-expressing any of the non-functional insertion mutant channels.

**Figure 5:**
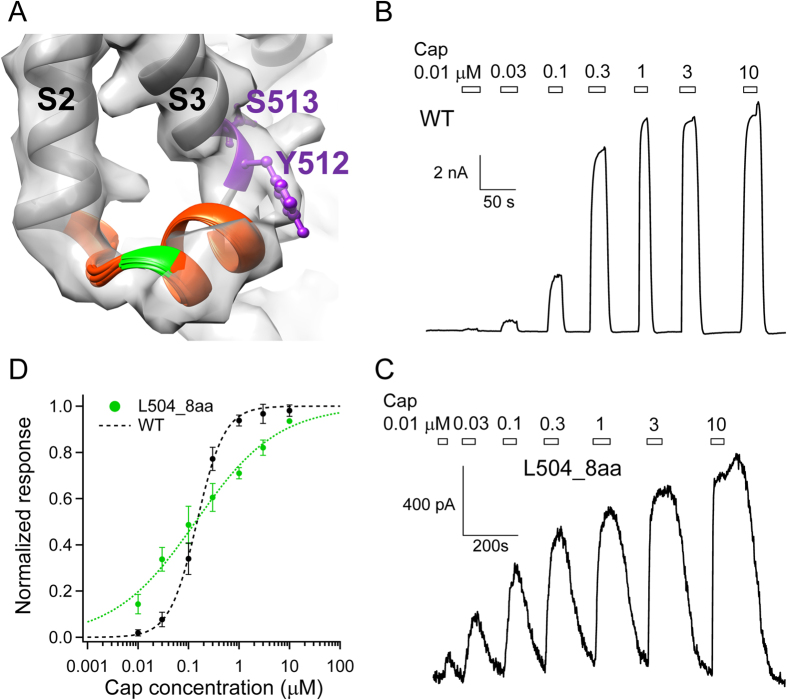
Peptide insertion before the S2-S3 linker does not disrupt capsaicin activation. (**A**) Structural models of the S2-S3 linker (orange) superimposed on the electron density map of TRPV1 in the apo state (EMD-5778). The position of L504 is highlighted in green; the side-chain of Y512 and S513 are shown in ball-and-stick format (purple). (**B**,**C**) Representative current trace of WT and L504_8aa mutant channel in response to increasing concentrations of capsaicin, respectively. (**D**) The concentration-response relationship of L504_8aa is fitted to a Hill function with EC50 of 0.19 ± 0.08 μM and slope factor of 0.6 ± 0.05 (n = 3). The wild-type TRPV1 curve (black dashed curve) is shown as comparison.

Interestingly, cells expressing each of the functional insertion mutants exhibited a fluorescence increase very similar in time course to that of the wild-type channels (Fig. 4). These observations indicated that unstructured peptide insertions in these mutant channels—all at the interface between the transmembrane domain and the intracellular domains—had a minor effect on capsaicin activation. This is particularly interesting for L504_8aa, which contains an eight-amino-acid peptide insertion at the S2-S3 linker just a few amino acids upstream from Y512 and S513, two key residues whose mutations are known to substantially affect capsaicin activation^14^,^16^. Because the structure of the S2-S3 linker was not determined in the original cryo-EM study^22^, we used Rosetta-based structural modeling to predict its likely structure. Our result suggested that this region forms a short helix, which is consistent with the recently available TRPV1 cryo-EM structure at a higher resolution^40^, with Y512 and S513 residing at its junction with S3 (Fig. 5A). In this structural model, the peptide-insertion site is also a flexible junction between two helical segments, S2 and the S2-S3 linker. Patch-clamp recordings revealed that L504_8aa responded to capsaicin with an EC50 value similar to that of the wild-type channel (WT: 0.15 ± 0.02 μM. L504_8aa: 0.19 ± 0.08 μM. *P* = 0.57) (Fig. 5B to D). Taken together, results from our functional and computational analyses are consistent with the hypothesis that the S2-S3 linker is likely a mobile structure. The anticipated mobility would facilitate the swinging motion of Y512 upon capsaicin binding towards the nearby ligand-binding pocket^14^,^16^,^23^.

We further observed normal and robust fluorescence signal from cells expressing the wild-type channel or any of the three functional insertion mutants when extracellular proton or divalent cation Mg^2+^ was used as activator (Fig. 4). No detectable difference in the time course of fluorescence signal was observed in these recordings in comparison to capsaicin-induced signals. H^+^ and Mg^2+^ are expected to directly interact with aqueous-accessible channel structures on the extracellular side. Our observations indicate that, like capsaicin, proton- and divalent cation-induced activation conformational changes can tolerate insertions at the interface between the intracellular domains and the transmembrane core domain.

### The N-terminal insertion in F430_3aa disrupted Ca^2+^-dependent desensitization

**Figure 6:**
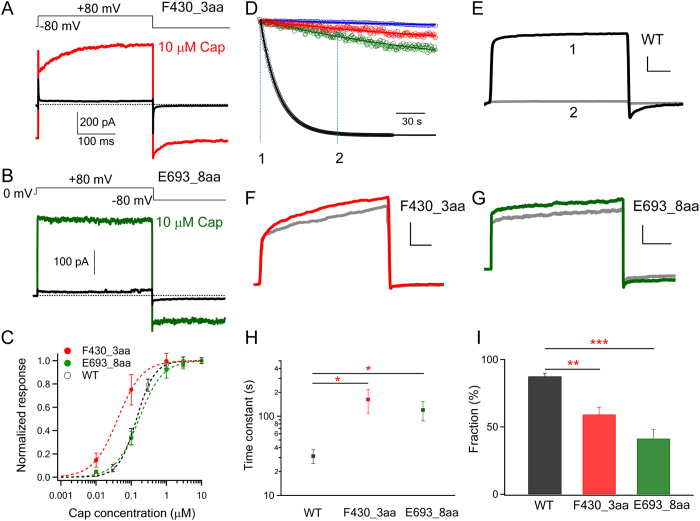
Short peptide insertions significantly impaired the coupling between the intracellular domains and the transmembrane core domain. (**A,B**) Representative traces of F430_3aa and E693_8aa activated by capsaicin. (**C**) Capsaicin concentration-response curves of WT (EC50: 0.15 ± 0.02 μM; slope factor: 1.82 ± 0.22), F430_3aa (EC50: 0.07 ± 0.03 μM; slope factor: 1.39 ± 0.38) and E693_8aa (EC50: 0.26 ± 0.06 μM; slope factor: 1.10 ± 0.23). (n = 3–4). (**D**) Representative whole-cell patch recordings (with 2 mM Ca^2+^ in solutions) of WT TRPV1 (black), F430_3aa (red) and E693_8aa (green) activated by 1 μM capsaicin and desensitized through Ca^2+^-dependent desensitization mechanisms. WT TRPV1 exhibited little desensitization in the absence of Ca^2+^ in the solution (blue). Current decay over time from the capsaicin-activated peak current was superimposed with an exponential fit (smooth curve). (**E** to **G**) Raw current traces of each channel at time point 1 (colored) and 2 (grey). The X- and Y-axis scales are 40 ms and 1 nA respectively. (**H**) Comparison of current decay time constants. *, p < 0.05. (**I**) Averaged fraction of desensitization, calculated as (I_peak_ − I_desensitized_)/I_peak_ *100, where I_peak_ is the peak current amplitude potentiated by 1 μM capsaicin and I_desensitized_ is the current level when current decay caused by desensitization has reached steady-state. **, p < 0.01; ***, p < 0.001.

A possible explanation for the lack of difference in fluorescence signal between wild-type and mutant channels might be insensitivity of the non-quantitative optical method to subtle changes. Therefore, we examined the functional mutant channels with patch-clamp recordings, which confirmed observations from fluorescence recordings. We found that F430_3aa produced rapid capsaicin-induced current responses just like the wild-type channel (Fig. 6A,C). Extended recordings, however, revealed that there was a substantial difference in the current decline phase. The current decline represents Ca^2+^-dependent acute desensitization, a process thought to be mediated by multiple mechanisms, including binding of Ca^2+^-calmodulin to the ankyrin-like repeat domain^34^,^35^,^41^,^42^, phosphorylation^43^, and binding of PIP2 to the C-terminus^44^,^45^,^46^,^47^,^48^, that allosterically modulate the stability of the open pore conformation. For the wild-type channel, as expected little desensitization was observed in the absence of Ca^2+^. With 2 mM Ca^2+^ present in the solution, desensitization proceeded with a time constant of 31.4 ± 6.2 s (n = 6). In comparison, the current decline of F430_3aa channels proceeded slowly, with a time constant of 162.4 ± 53.5 s (n = 5; p < 0.05). As a result, most of the current remained at the end of the 3 min recording (Fig. 6D to I). Therefore, insertion of an unstructured peptide at the junction between the N-terminus and the transmembrane domain weakened functional coupling between these domains, substantially limited the ability of intracellular Ca^2+^-mediated events to modulate gating of the channel pore.

### The C-terminal insertion in E693_8aa also disrupted Ca^2+^-mediated desensitization

Patch-clamp recordings showed that E693_8aa exhibited capsaicin-dependent activation similar to that of the wild-type and F430_3aa channels. However, like F430_3aa, we found that the desensitization process of E693_8aa was also substantially slower compared to the wild-type channel (Fig. 6). The time constant for E693_8aa desensitization was estimated to be 119.8 ± 32.7 s (n = 4), which is significantly larger than that of the wild-type channel (p < 0.05). Therefore, inserting an unstructured peptide at the junction between the C-terminus and the transmembrane domain also weakened functional coupling between these domains.

### Heat activation of N- and C-terminal insertion mutants

**Figure 7:**
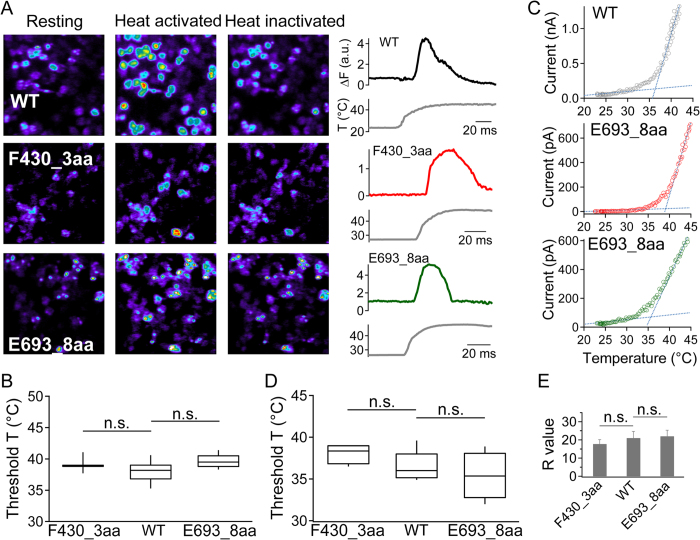
Heat activation of insertion mutants. (**A**) Representative calcium imaging of WT, F430_3aa and E693_8aa with corresponding fluorescence signal intensity traces. Increased calcium influx was observed upon channel activation by temperature changes. Due to inactivation, intracellular calcium level dropped after the peak even at persistent high temperature. (**B**) Box-and-whisker plot of heat activation threshold temperature of WT and insertion mutants. The threshold temperature in imaging studies was defined as the temperature at the starting point of the rapid raising phase of the fluorescence signal. The whisker top, box top, line inside the box, box bottom, and whisker bottom represent the maximum, 75^th^ percentile, median, 25^th^ percentile, and minimum value of each pool of measurements, respectively. (**C**) Representative heat activation of WT and insertion mutants by patch-clamp recording at +80 mV. Two linear functions (blue dotted lines) were fitted to determine the heat activation threshold temperature. (**D**,**E**) Comparison of the threshold temperature and the R value of WT and insertions mutants. n.s., not statistically significant by *t*-test; *n* = 4–8.

TRPV1 is a prototypical heat-sensing ion channel^1^; however, how heat is sensed and used to promote activation conformational change remains unclear. Both N- and C-terminal regions, as well as extracellular regions, have been previously suggested to mediate heat activation^10^. If the structural element(s) responsible for high-sensitivity heat activation resides in the N- or C-terminal domain, F430_3aa and E693_8aa would offer an opportunity to test the coupling of heat-sensing and pore opening events. Indeed, for the TREK-1 channel a triple-glycine insertion that decoupled C terminus was found to largely reduce temperature sensitivity^28^. We first measured heat response of F430_3aa and E693_8aa with live-cell calcium imaging (Fig. 7A). Cells expressing the wild-type channel, F430_3aa, or E693_8aa exhibited clear increase in fluorescence intensity when temperature rose above similar threshold values (Fig. 7B. wild-type: 38.1 ± 0.3 °C, n = 58; F430_3aa: 39.2 ± 0.2 °C, n = 36; E693_8aa: 39.7 ± 0.2 °C, n = 28). Upon continuous heating, the fluorescence signal exhibited characteristic transient nature for all three channel-types.

To better quantify temperature sensitivity of F430_3aa and E693_8aa, we performed patch-clamp recordings. In close agreement with observations from fluorescence imaging, we observed typical heat-induced current from both mutants (Fig. 7C). Their heat activation threshold values calculated from current recordings (F430_3aa: 38.1 ± 0.6 °C, n = 4; E693_8aa: 35.4 ± 1.4 °C, n = 4) were again similar to that of the wild-type channel (36.6 ± 0.6 °C, n = 9) (Fig. 7D). The sensitivity of heat response, reflected by the steepness of current increase, was quantified by the R-value^30^ (see Materials and Methods for details). We found that the R-value also exhibited no significant difference between the wild-type and two insertion mutants (Fig. 7E). These observations demonstrated that insertion of an unstructured peptide between the intracellular and transmembrane domains did not prevent heat activation (even though they substantially disrupted Ca^2+^-dependent desensitization). We did observe that the relative amplitude of heat response compared to capsaicin response was reduced in the mutant channels (wild-type: 87 ± 10%, n = 6; F430_3aa: 37 ± 2%, n = 3; E693_8aa: 40 ± 6%, n = 3). Because capsaicin activates wild-type mTRPV1 to a stable open probability near 100%^30^, such reduction in relative amplitude indicated that the heat-activated pore conformation of the mutant channels might be less stable when allosteric coupling between transmembrane and intracellular domains was disrupted.

## Discussion

Unstructured peptide-insertion screening, though admittedly a coarse-grained test, allowed for a quick survey of the TRPV1 intracellular structures when guided by the high-resolution cryo-EM structures. We found that all functional mutants have an insertion at the “joint” between rigid helical structures. In addition, these are also locations that appear to be capable of spatially accommodating the additional mass of the inserted unstructured peptide, a point highlighted by the fact that an eight-amino-acid insertion between E693 and S694 yielded a functional channel (E693_8aa), while increasing the size of inserted peptide to 12 amino acids was not tolerated. Exceptions to this general pattern are the two insertions bracketing the N-terminal β-hairpin structure, where structural flexibility and spatial accommodation were anticipated. The observation hence lends support of the notion that the β-sheet formed jointly by the N- and C-terminal segments may mediate important subunit-subunit interactions^22^. Furthermore, all insertions in the middle of helical segments, e.g., the S4-S5 linker or S6, yielded non-functional channels. Noticeably, only two of the non-functional mutants (H365_3aa and Q561_4aa) appeared to be due to trafficking defects, highlighting the sensitivity of TRPV1 gating to structural integrity of the intracellular domains. Therefore, the fact that two functional insertion mutants, F430_3aa and E693_8aa, at the junctions between the intracellular domains and the transmembrane core domain exhibited selective defects in gating (Ca^2+^-dependent desensitization but not activation) is worth noting.

Among TRPV1 stimulations, the capsaicin-channel interaction is the best-understood case in both location and detailed atomic interactions^14^,^15^,^16^,^17^,^18^,^19^. None of the functional insertion mutations disrupted capsaicin activation, in agreement with the notion that capsaicin-activation machinery mostly resides within the transmembrane region. Other two modalities tested in this study, Ca^2+^ and proton, are less well defined and likely work through multiple mechanisms (for Ca^2+^) or targets (for proton); nonetheless, their effects clearly start from the intracellular and extracellular side, respectively, which explains our opposite experimental observations.

In comparison to the chemical stimulations, physical stimulations for TRPV1—voltage, mechanical force, and heat—are still very poorly understood. The situation motivated the search in recent years for chemical stimuli that activate TRPV1 through the heat activation pathway. Candidates identified so far include divalent cations such as Mg^2+^ and Ba^2+ ^49,50, Na^+ ^51, DkTx^21^ and RhTx^6^. While drastically different in size, all of these modulators are found to be effective only when applied from the extracellular side, with their likely binding sites clustered at the outer pore region. We found in the present study that extracellular Mg^2+^-induced activation was only slightly disturbed in the functional insertion mutants, providing new supportive evidence that Mg^2+^ promotes heat activation by interacting with TRPV1 structure(s) accessible from the extracellular side.

The structural and mechanistic bases for heat activation of TRPV1 and related TRP channels remain to be explored. Noticeably, recent studies using centipede and spider toxins^6^,^21^, Mg^2+ ^49,50, and Na^+ ^51 provided fresh evidence that, together with existing findings (summarized in ref. 52), suggests that the outer pore region is involved in the heat activation process. In the present study, we found that F430_3aa and E693_8aa, with almost completely decoupled Ca^2+^-dependent desensitization, exhibited nearly normal heat activation. Noticeably, both capsaicin- and heat-induced activation in these mutant channels appeared to be less stable than that of the wild-type channel, which may be due to weakened allosteric coupling between the intracellular domains and the transmembrane core domain. Our observations lend further support to the idea that the transmembrane core domain is crucial for heat activation. In addition, they argue against the scenario in which an intracellularly located heat sensor is solely responsible for driving heat activation of TRPV1.

As an allosteric protein, TRPV1’s activation process can be dynamically modulated by numerous factors. Among them, Ca^2+^ ions are important because not only the effects are substantial and rapid but also Ca^2+^ influx through activated channels provides a powerful physiological negative feedback mechanism^10^. Modulation of channel activity by intracellular Ca^2+^ is mediated by multiple mechanisms utilizing calmodulin^34^,^35^,^41^,^42^, Phosphorylation^43^, and PIP2^44^,^45^,^46^,^47^,^48^. As F430_3aa and E693_8aa exhibit specific and substantial deficits in Ca^2+^-dependent desensitization, they may serve as useful tools to further investigate the allosteric coupling process between the intracellular domains and the transmembrane core domain.

## Additional Information

**How to cite this article**: Ma, L. *et al*. Exploring functional roles of TRPV1 intracellular domains with unstructured peptide-insertion screening. *Sci. Rep.* **6**, 33827; doi: 10.1038/srep33827 (2016).
